# The Southern Medical College Association

**Published:** 1892-12

**Authors:** 


### THE SOUTHERN MEDICAL COLLEGE ASSOCIATION.

At the recent meeting of the Southern Surgical and Gynecological Association in Louisville representatives of the different Southern Medical Colleges organized the above Association, and adopted the following:

### RULES AND REGULATIONS FOR THE ORGANIZATION AND MAINTENANCE OF THE SOUTHERN MEDICAL COLLEGE ASSOCIATION.

This Association shall be composed of delegates from Southern medical colleges, whose faculties have signified a desire to become members thereof, signed these rules of organization, and paid the membership fee of $5.00.

The objects of the Association are to cultivate closer and more intimate relations between medical colleges, and to elevate the standard of medical education by requiring a more thorough preliminary training and an increased length of medical study.

The Association shall be composed of one or more delegates from each medical college belonging thereto, who shall be elected annually by their respective faculties. Each college shall be entitled to one vote in the transactions of the Association.

The officers shall consist of a President, Vice-President, Secretary and Treasurer, who shall be elected annually, just before the adjournment of the annual meetings, and shall perform the respective duties pertaining to these offices in similar organizations.

The meetings of the Association shall be held at the same time and place of the meetings of the Southern Surgical and Gynecological Association, unless otherwise determined by the Association.

### REQUIREMENTS FOR MATRICULATION.

Every student applying for matriculation must possess the following qualifications:

He must hold a certificate as the pupil of some known reputable physician, showing his moral character and general fitness to enter upon the study of medicine.

He must possess a diploma of graduation from some literary or scientific institution of learning, or certificate from some legally constituted high school,

General Superintendent of State Education or Superintendent of some county Board of Public Education, attesting the fact that he is possessed of at least the educational attainments required of second grade teachers of public schools ; *provided, however*, that, if a student so applying is unable to furnish the above and foregoing evidence of literary qualifications, he may be permitted to matriculate and receive medical instructions as other students, and qualify himself in the required literary departments, and stand his required examination as above specified, prior to offering himself for a second course of lectures.

The foregoing diploma or certificate of educational qualifications, attested by the Dean of the medical college attended, together with a set of tickets showing that the holder has attended one full course of medical lectures, shall be essential to attendance upon a second course of lectures in any college belonging to this Association.

BRANCHES OF MEDICAL SCIENCE TO BE INCLUDED IN COURSE OF INSTRUCTIONS.

Anatomy, Physiology, Chemistry, Materia Medica and Therapeutics, Theory and Practice of Medicine, Pathology, Surgery, Obstetrics and Gynecology, Hygiene, Medical Jurisprudence (Forensic Medicine) and Special Laboratory work, as hereafter provided.

QUALIFICATIONS FOR GRADUATION.

Candidates for graduation in addition to the usual requirements of medical colleges, must have attended three courses of lectures of not less than six months each in three separate years.

Must have dissected in two courses, and attended two courses of clinical or hospital instructions.

And must have attended one course in each of the separate laboratory departments, to-wit:

1. Histology and Bacteriology.
2. Chemistry.
3. Operative Surgery.

These requirements shall not apply to any student who has received a course of medical lectures prior to September 1, 1893.

COMMITTEE.

W. T. BRIGGS,
J. B. MARVIN,
J. S. CAIN.

The..................................of..........................................agrees to the above regulations and thereby becomes a member.

